# Identification of novel microRNAs in the Verticillium wilt-resistant upland cotton variety KV-1 by high-throughput sequencing

**DOI:** 10.1186/2193-1801-3-564

**Published:** 2014-09-27

**Authors:** Xiaohong He, Quan Sun, Huaizhong Jiang, Xiaoyan Zhu, Jianchuan Mo, Lu Long, Liuxin Xiang, Yongfang Xie, Yuzhen Shi, Youlu Yuan, Yingfan Cai

**Affiliations:** State Key Laboratory of Cotton Biology, Henan Key Laboratory of Plant Stress Biology, College of Life Sciences, Henan University, Kaifeng, 475004 China; College of Bioinformation, Chongqing University of Posts and Telecommunications, Chongqing, 400065 China; State Key Laboratory of Cotton Biology, Cotton Institute of the Chinese Academy of Agricultural Sciences, Key Laboratory of Cotton Genetic Improvement, Ministry of Agriculture, Anyang, Henan 455000 China

**Keywords:** *Gossypium hirsutum*, KV-1, *Verticillium dahliae Kleb*, Deep sequencing, MicroRNAs

## Abstract

Plant microRNAs (miRNAs) play essential roles in the post-transcriptional regulation of gene expression during development, flowering, plant growth, metabolism, and stress responses. Verticillium wilt is one of the vascular disease in plants, which is caused by the *Verticillium dahlia* and leads to yellowing, wilting, lodging, damage to the vascular tissue, and death in cotton plants. Upland cotton varieties KV-1 have shown resistance to Verticillium wilt in multiple levels. However, the knowledge regarding the post-transcriptional regulation of the resistance is limited. Here two novel small RNA (sRNA) libraries were constructed from the seedlings of upland cotton variety KV-1, which is highly resistant to Verticillium wilts and inoculated with the V991 and D07038 *Verticillium dahliae* (*V. dahliae*) of different virulence strains. Thirty-seven novel miRNAs were identified after sequencing these two libraries by the Illumina Solexa system. According to sequence homology analysis, potential target genes of these miRNAs were predicted. With no more than three sequence mismatches between the novel miRNAs and the potential target mRNAs, we predicted 49 target mRNAs for 24 of the novel miRNAs.

These target mRNAs corresponded to genes were found to be involved in plant–pathogen interactions, endocytosis, the mitogen-activated protein kinase (MAPK) signaling pathway, and the biosynthesis of isoquinoline alkaloid, terpenoid backbone, primary bile acid and secondary metabolites. Our results showed that some of these miRNAs and their relative gene are involved in resistance to Verticillium wilts. The identification and characterization of miRNAs from upland cotton could help further studies on the miRNA regulatory mechanisms of resistance to Verticillium wilt.

**Electronic supplementary material:**

The online version of this article (doi:10.1186/2193-1801-3-564) contains supplementary material, which is available to authorized users.

## Introduction

Cotton is a fiber crop with worldwide economic importance. It is vulnerable to some diseases such as Fusarium wilt and Verticillium wilt, which caused by the phytopathogenic fungus *V. dahliae Kleb* and could have severe detrimental effects on the cotton in China, the Americas, and Mediterranean regions (Cai et al. 2009; Gao et al. 2010). The soil-borne fungal pathogen has displayed extraordinary genetic plasticity and broad-hosts rang in diverse ecological niches (Fradin & Thomma 2006). They invade the vascular tissue by invading cortex of root via wounds or epidermal cells to cross the endodermis. The plant could show leaf vein browning, wilting, yellowing, vascular discoloration, defoliation and death after conidia are produced in large numbers and migrate via the xylem to the aerial part of the plants (Klosterman et al. 2009; BejaranoAlcazar et al. 1997). Consequently, Verticillium wilt is often referred to as the ‘cancer of cotton crops’. Therefore, the control against Verticillium wilt is necessary. However, it is also particularly difficult. The *V. dahliae* strains D07038 and V991 were used in this study and are moderately virulent and virulent, respectively. At the peak of infection, the defoliating type V991 strain has a disease incidence of 99.10% and a disease index of 81.98 relative to uninfected control plants (Fan & Jiang 2006). The moderately toxic and defoliating wild-type D07038 strain was obtained from infected *Gossypium hirsutum*, plant and had a mean disease index of 46.1%.

Cotton varieties are assigned to five disease-resistance classes based on relative disease index scores. Varieties with relative disease index scores of 0.0 are designated immune (I); scores between 0.1 and 10.0 indicate high resistance (HR); varieties with scores between 10.1 and 20.0 are resistant (R); scores between 20.1 and 35.0 indicate tolerant (T) varieties; and varieties with scores greater than 35.0 are susceptible (S) (Zhang et al. 2012a). The cotton variety Zhongzhimian KV-1 (KV-1) was developed at Institute of Plant Protection, Chinese Academy of Agricultural Sciences (Beijing, China) and shows stable high resistance to Verticillium wilt (Zhao et al. 2009) with no significant differences between relative disease indices over three consecutive years. In this study we identify the novel miRNAs by high-throughput sequencing applying KV-1 highly resistant to Verticillium wilt.

MicroRNAs are small (~21 nt), single strain and non-coding RNAs which play key role in plant developmental and responses to stress. Precursor stem-loop secondary structures are characteristic features of miRNAs and are conserved across species (Bartel 2004; Carrington & Ambros 2003). miRNAs are transcribed from DNA. However, those miRNAs are not translated into protein. They regulate functions of other genes at the level of protein synthesis. To date, 4,933 miRNAs which are from 53 eudicotyledon species had been identified and submitted to miRBase (miRBase Release 20.0, http://www.mirbase.org/). A total of 78 precursors and 80 mature *Gossypium hirsutum* miRNAs have been deposited in miRBase. Previous studies have focused primarily on miRNA expression in ovules and during fiber development. Knowledge of miRNA regulatory mechanisms associated with *G. hirsutum* resistant to Verticillium wilt is very limited. The miRNAs, miR1321-1334, were predicted and identified in cotton using bioinformatics and the Solexa sequencing method (Yin et al. 2012a). Identification of novel miRNAs provides us an important genomic resource to investigate of the miRNAs regulatory mechanism of the resistance to wilt disease in upland cotton.

This study aimed to identify novel miRNAs and their potential target genes, which specifically expressed in the Verticillium wilt-resistant upland cotton variety KV-1. So two independent small RNA (sRNA) libraries were constructed from whole KV-1 seedlings inoculated with the V991 and D07038 *V. dahliae* strains; the libraries were then sequenced using the Illumina Solexa system. Then the selected upland cotton miRNAs were confirmed by quantitative real time PCR (qRT-PCR).

The potential target genes of these novel miRNAs were predicted by transcriptome sequencing in *G. hirsutum*. We found some of these targets could function as new transcripts involved in plant-pathogen interactions, which will facilitate identification of candidate genes *in G. hirsutum* for resistance to *V. dehliae*.

## Materials and methods

### Fungal strains and inoculums preparation

The wild-type pathogenic *V. dehliae* V991 strain are used for inoculations, which was isolated from an infected upland cotton plant. Compared with the intermediately aggressive D07038 strain obtained from the Cotton Research Institute of the Chinese Academy of Agricultural Sciences, V991 is highly toxic, defoliant and more virulent. For conidial production, V991 and D07038 were subcultured from potato dextrose agar plates onto Czapek’s medium and incubated at 26°C for 7 days. Fungal cultures were filtered through sterile gauze to removal the mycelium. The concentrations of the conidial suspensions were counted under a microscope using a hemocytometer and adjusted to 1.0 × 10^7^conidia/ml with sterile distilled water (Zhang et al. 2012b).

### Plant culture and treatment

To investigate the mechanism of disease resistance in the cotton variety Zhongzhimian KV-1, we infected seedlings with the defoliating *V. dahliae* strains D07038 (moderate virulence) and V991 (high virulence). Seeds were treated with 98% H_2_SO_4_ to remove the surface fuzz, and then soaked in 70% ethanol for 5 min and in 10% H_2_O_2_ for 1 h to sterilize the surface, followed by three rinses with sterile water. Surface-sterilised seeds were germinated at 26°C on plates containing sterilised water and filter paper. The seedlings were grown under supplemental light outside the laboratory for 2 days and then transferred to an autoclaved mixture of vermiculite and peat (1:1 v/v). Cotton seedlings were inoculated using a root dip method. The *V. dahliae* strains D07038 and V991 were cultured for 7 days and used separately to infect roots of 21-day-old seedlings, at which point the seedlings had grown one to two euphylla. Roots were infected with *V. dahliae* inoculums of 10^7^ CFU/mL (10 mL) fungal suspensions and incubated for ~1–2 days at 26°C and ~80% humidity. The seedlings were treated for 24 and 48 h with D07038 as the control group or with V991 as the experimental group. Total RNAs from seedlings were harvested 24 and 48 h after inoculation and pooled in a 1:1 ratio. The whole seedling was chosen as the experimental material because the pathogenic fungus directly infects cotton roots in soil and enters the vasculature through the cortical cells, resulting in disease in the stems and leaves (Cai et al. 2009).

### Construction and sequencing of sRNA libraries

Two sRNA libraries were constructed using the procedure of Kwak et al. (Kwak et al. 2009). Total RNA was isolated from *G. hirsutum* seedlings using a Plant RNA Kit (Watson Biotech, Shanghai, China) according to the manufacturer’s instructions. All the RNA samples were quantified and equalised to guarantee equal amounts of RNA from each treatment. The quality of RNA samples was evaluated using an Agilent 2100 Bioanalyzer (Agilent, Waldbronn, Germany). All the RNA samples were separated by 15% urea denaturing polyacrylamide gel electrophoresis and the 18–30 nucleotide sRNA bands were excised and extracted with 0.4 M NaCl over night at 4°C. The isolated sRNAs were converted into DNA by reverse transcription PCR (RT-PCR) after being ligated sequentially to 5′ and 3′ adapters. The DNAs were confirmed by sequencing with Solexa sequencing technology (BGI, Shenzhen, China).

### Bioinformatics analysis

Raw sequences were processed using the SOAP 2.0 software (BGI, Shenzhen, China) as reported previously (Li et al. 2008). Without the vector sequences, the sequences longer than 17 nt were considered for further analyses. Excluding rRNA, scRNA, snoRNA, snRNA, tRNA and the sequences containing a polyA tail, the other sequences were compared with *G. hirsutum* non-coding RNA sequences in the NCBI GenBank and Rfam 10.1 databases. To identify conserved miRNAs in upland cotton, the unique small RNA sequences were selected to carry out BLASTN search in the miRBase. Conserved miRNAs require perfect match. The novel miRNAs are predicted according to the characteristic hairpin structure of microRNA precursors by the RNA-folding software Mireap (http://sourceforge.net/projects/mireap/), which could provide the information about the secondary structures, the minimum free energies and the Dicer cleavage sites of the unidentified sRNA tags. We used the *G. raimondii* genome sequences (ftp://ftp.ncbi.nih.gov/pub/TraceDB/gossypium_raimondii/) as an miRNA positioning reference sequence. Only mature miRNA sequences were considered, which locatein the stem region of the stem-loop structure and range between 20–22 nt as well as a minimal folding free energy index (MFEI) greater than 0.85. After the selected sequences by the Mireap were folded into the secondary structure, a novel miRNA was selected when it was at 5′end arm of the stem of a perfect stem-loop structure, and another sequence was at 3′end arm and then the small RNA was consisted as a novel miRNA from cotton.

The targets of these novel microRNAs were predicted through BLAST analysis of transcriptome sequencing data from KV-1 plants infected with *V. dahliae Kleb*. Target gene predictions followed the methods reported by Allen et al. (Allen et al. 2005). Sequences less than 4 nt mismatches relative to the query miRNA sequences were chosen manually. We allowed one mismatch in the region complementary to nucleotide positions 2–12 of the miRNA except the mismatch at a predicted cleavage site at positions 10 or 11. We also allowed three additional mismatches between positions 12 and 22 except more than two continuous mismatches. The G:U base pair was treated as a mismatch with a value of 0.5.

### Confirmation of predicted miRNAs by qRT-PCR

To confirm miRNA expression, we analyzed seven conserved miRNAs by RNA-tailing and primer-extension qRT-PCR. Small RNA was separated from seedlings by the RNAiso for sRNA reagent (TaKaRa, Dalian, China). According to the manufacturer’s protocols (New England Biolabs, Beijing, China), the treated sRNA (1.5 μg) was polyadenylated by polyA polymerase (PAP) at 37°C for 30 min of the Poly(A) Tailing Kit (New England Biolabs). The RNAs were dissolved in diethylpyrocarbonate (DEPC)-treated water and reverse-transcribed with 200 U M-MLV reverse transcriptase (RNase Hˉ) and 2 μL 10 pmol poly(T) adapter after phenol-chloroform extraction and ethanol precipitation (Table 1) according to the reverse transcriptase M-MLV manufacturer’s instructions (TaKaRa) (Shi & Chiang 2005). Here *G. hirsutum* 5S ribosomal RNA (rRNA, GenBank: U32085.1) worked as the internal reference gene of PCR quantization. A 3′ adapter primer worked as the reverse primer for the miRNAs and the 5S rRNA. The sequence of the forward primer was selected by the entire test miRNA sequence. Reverse transcription reactions were performed at 50°C for 1 h, then inactivated at 75°C for 15 min. The qRT-PCR was carried out with SYBR Premix Ex Taq II (TaKaRa, Dalian, China) on a IQ™^5^ real-time PCR detection system (Bio-Rad). Each PCR included 0.5 μL template cDNA, approximately 100 pg sRNA, 5 μL 2× SYBR Green II PCR master mix, and 0.5 μL 10 pmol the forward and reverse primers (Table 1) in 10 μL reaction system. All reactions were performed in triplicate for each sample. All reactions used the following thermal cycling profile: an initial step at 95°C for 60 s, followed by 45 cycles of amplification (95°C for 20 s, 58°C for 20 s, 72°C for 30 s) with a termination reaction at 4°C. Relative miRNA expression levels were calculated by the method of Livak and Schmittgen (Livak & Schmittgen 2001).

**Table 1:** **Primers Used in this Study**

Name	Sequence(5′ → 3′)
miR5266	CTGGGGGACTGTCTGGGGC
miR5562	TGTGGAGTCTTTTGCATGAAG
miR3444a-5p	TTGGGAGCTCGATGAGATCG
miR2867-5p	TGTGCCATCCCACACATC
miR1148	CCAACGTGCAGGGGGACA
miR1423a-5p	AGGCAACTACACGTTGGGCG
miR952a	AACAGAGCATGCCGTTGGT
Ghr5S RU	GGATCCCATCAGAACTCCAC
Ghr5S RL	ACGAGGACTTCCCAGAAGGT
Poly(T) adapter	GCTGTCAACGATACGCTACGTAACGGCATGACAGTG(T)22V
Reverse primer	GCTGTCAACGATACGCTACGTAACG
*V = A, G, C;	

## Results

### Analysis of sequences from libraries of small RNAs

Totally 2 small RNA, namely sRNA libraries were constructed and sequenced to identify sRNAs from *G. hirsutum* L. seedlings infected with either of two strains of *V. dahliae*. The *V. dahliae* strains D07038 and V991 were cultured separately for 7 days on infected 21-day-old KV-1 cotton seedlings. Then roots were infected with the cultured D07038 or V991 as control and experimental treatments, respectively. Two sRNA cDNA libraries were generated from pooled total RNA isolated 24 and 48 h after inoculation for each treatment. Each library was sequenced by a Solexa/Illumina analyser and generated 14,491,706 primary reads for KV-1 plants infected with D07038, and 14,543,279 primary reads for KV-1 plants infected with V991 (Table 2).

**Table 2:** **Statistics of small RNA sequences from two upland cotton libraries generated from KV**-**1 seedlings infected with the D07038 or V991 strain**

Category	KV-1_D07038		KV-1_V991	
	Sequences	Unique sequences	Sequences	Unique sequences
Clean reads	14,156,228	4,348,363	14,186,947	4,597,927
miRNA	3,689,747	34,177	2,956,044	27,783
rRNA	745,970	79,074	1,549,145	145,979
snRNA	8,674	2,586	12,067	3,710
snoRNA	2,518	953	4,612	1,429
tRNA	232,045	13,306	778,699	40,648
Unannotated	9,477,274	4,608,248	8,886,380	4,378,378

**Figure 1:**
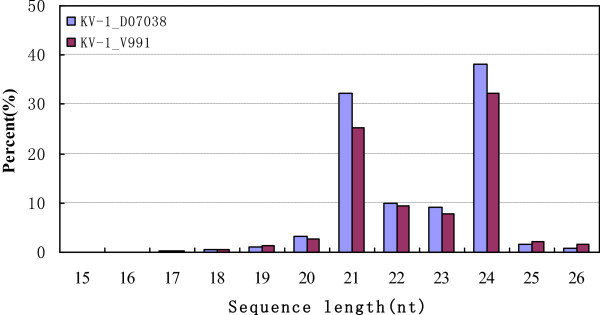
**The size distribution of the small RNAs in upland cotton seedling KV-1_D07038 and KV-1_V991 libraries.**

Approximately 75–90% of the sRNAs in the libraries were 20–24 nt in length, and 21 and 24 nt are the main size groups (Figure 1). This distribution of sizes was similar to the other plants species and indicates that cotton miRNAs are primarily processed by DCL1 (Wang et al. 2007). Most of the sRNAs in the libraries were 24 nt long and were 38.2% and 32.3% of the total number of sequences from the D07038 and V991 libraries, respectively. Small RNAs that were 21 nt long made up 32.3% and 25.2% of the D07038 and V991 libraries, respectively, and 22 nt sRNAs made up 9.8% and 9.5% of the D07038 and V991 libraries, respectively. This observation pointed out could reflect the complexity of the cotton genome because 24 nt sRNAs are reported to regulate the heterochromatin modification of the genomes with many repetitive sequences (Vazquez 2006). The sRNAs identified by deep sequencing represented almost all RNA classes. By comparing with the sequences to those in databases, most of our sRNAs were assigned to the different classes. The sequences that could not be assigned were used to perform the prediction of novel miRNAs by Mireap software.

Thirty-four miRNAs were screened out from 31 families. We studied the miRNAs from known families and found they showed significant difference. The abundance of individual miRNAs in the libraries varied drastically in our dataset (Table 3). Some miRNAs were represented only a few times while others existed in hundreds of thousands of copies, which indicated that the cotton seedlings contained a large and diverse collection of sRNAs. Among them, ghr-miR394 and ghr-miR2950 miRNAs showed significant differences between treatment and control groups. These observations indicate that different members could have different expression levels from the same miRNA family. Probably expression is specific to different tissues or specific to different developmental stages (Chi et al. 2011). The differential expression of a few conserved miRNAs in the two samples is not more than two times, means that several families involved in the development regulation of upland cotton seedlings.

**Table 3:** **Expression levels of upland cotton miRNA families showed by Solexa sequencing**

Family	KV-1_D07038 expressed	KV-1_V991 expressed	fold-change*	Family	KV-1_D07038 expressed	KV-1_V991 expressed	Fold-change*
ghr-miR156	678465	372468	-0.87	ghr-miR398	6	2	-1.59
ghr-miR162	587	727	0.31	ghr-miR399	23	26	0.17
ghr-miR164	17299	26429	0.61	ghr-miR479	135	195	0.53
ghr-miR166	147030	125066	-0.24	ghr-miR482	1093	1101	0.01
ghr-miR167	967	2049	1.08	ghr-miR827	4191	3246	-0.37
ghr-miR172	1	2	1.00	ghr-miR2948	6453	2260	-1.52
ghr-miR390	35883	18183	-0.98	ghr-miR2949	2528	3049	0.27
ghr-miR393	18	30	0.73	ghr-miR2950	6806	1028	-2.73
ghr-miR394	121	10	-3.60	ghr-miR3476	20464	16352	-0.33
ghr-miR396	3666	3444	-0.09				

*Compare the known miRNA expression level in two samples .The procedures are shown as following:

(1) Calibration of the transcript expression per million (TPM) by normalizing the expression of micoRNA in control and treatment samples.

Normalization formula: Normalized expression = Actual miRNA count/Total count of clean reads*1000000.

(2) Calculation of fold-change based on the normalized expression: Fold_change = log2 (KV-1_V991-std /KV-1_D07038 -std).

### Identification of conserved miRNAs from upland cotton

We raked through sequence homology analysis for known microRNAs in the two sRNA libraries with a requirement of at least 18 nt in length with a maximum of three mismatches compared to miRNAs in miRBase17. The abundance of specific miRNAs varied greatly between different miRNA families and between members within families. The depth of sequencing in our study was successfully identified 284 significant genes out of 443 differences expressed miRNA families of conservative miRNAs (Additional file 1). Among the miRNAs identified by comparing resistance to V991 and D07038 in KV-1 seedlings, 119 miRNAs were differentially up-regulated and 165 miRNAs were differentially down-regulated in cotton seedlings. As shown in Additional file 1, miR156, miR2911, miR2916, and miR172 were high expression in two sRNA libraries but a few conserved miRNAs (for example, miR161, miR163, and miR416) and most of the non-conserved miRNAs were found to have either very low expression. We also found some miRNAs such as miR5266, miR2867, miR3437, miR2086, and miR5652 were expressed preferentially corresponding to resistance to Verticillium wilt in KV-1 cotton. The results indicate that the expression of miRNAs was significantly affected by infection with *V. dahliae*, which suggested that miRNAs could be participate in the regulation of gene expression as part of the resistance to Verticillium wilt infection in the KV-1 variety.

### Identification of novel miRNAs in upland cotton

Many researchers have identified novel miRNAs in numerous plant species by high-throughput sequencing means based on small RNA. They have find sRNA to a genomic location based on genomic DNA sequences of the species to predict secondary structure characteristic of an miRNA precursor. As described by Reinhart et al. (Reinhart et al. 2002), the lowest-energy structures sRNA is identified as new miRNAs. A total of 78 miRNA precursors and 80 mature miRNAs from *G. hirsutum* are listed in miRbase release 20 and they belong to 51 miRNA families respectively. We screened the sRNA sequences generated in this study against the Rfam database to remove all known non-coding RNAs, which including scRNA, rRNAs, snoRNAs, tRNAs, piRNA, *et al*. For the identification of novel cotton miRNAs using the Mireap software, we used the *G. raimondii* genome sequence as an miRNA positioning reference sequence. We predicted 1141 miRNA candidate genes from the two sRNA libraries.

For 37 of the genes (Table 4), complementary miRNA* species (Meyers et al. 2008) with MFEI values higher than 0.85 were present in the libraries providing an indication of precise excision from the stem-loop precursor, which has recently been proposed as a primary criterion for the confident identification of an miRNA. The presence of microRNA* sequences is very important criterion because it indicates the release of the microRNA duplex from the predicted fold-back structure (Lu et al. 2006; Rajagopalan et al. 2006; Fahlgren et al. 2007), which were predicted using the genome sequence flanking the cloned sRNAs for all 37 of the novel microRNAs (Additional file 2) and microRNA precursors varied in length from 69 to 345 nt. Putative hairpin structures are shown in Additional file 3 with the novel miRNA candidates highlighted in blue. The predicted hairpins had MFEI ranging from 0.86 to 1.64 and negative folding free energies ranging from -117.6 to -33.8 kcal·mol^-1^. These values were much lower than the reported folding free energies of tRNA (-27.5 kcal·mol^-1^) and rRNA (-33 kcal·mol^-1^) (Bonnet et al. 2004).

**Table 4:** **Identified of novel miRNAs from upland cotton**

miRNA_id	Mature miRNA sequence(5′→3′)	LM(nt)	MFEIs
ghr-miR8156-5p	AAACUAUUCCUGGCUGAUUCG	21	1.00
ghr-miR7513-3p	AAUCAGCCAGGAAUCGUUUGA	21	0.97
ghr-miR8157-5p	AAGGCAAAGGAAGAAAAAGAGUG	23	1.08
ghr-miR8158-3p	AAGGGAGAACCUAGAUUCAUU	21	1.21
ghr-miR8159-5p	AAUGGAGGAGUUGGAAAGAUU	21	1.23
ghr-miR8160-5p	ACAGCUUUAGAAAUCAUCCCU	21	1.64
ghr-miR8161-3p	GUGGAUUAAAAUUUUGGUUGG	21	1.05
ghr-miR8162-5p	ACUUGCCUGCAUCUUUCAAAGA	22	0.87
ghr-miR8163-3p	GGUUGCUUACUUCUCUUCUGU	21	1.10
ghr-miR8164-3p	CCUAAUAAGGAUGAUGUCUCA	21	1.03
ghr-miR8165-3p	UCCAUAUUUCACUAUCUCUUA	21	1.31
ghr-miR8166-3p	CACAGGGACAAUACCUUCUAC	21	1.04
ghr-miR8167-5p	AGCUUUAGAAAUCAUCCCUU	20	1.54
ghr-miR7495a-3p	UUACUUUAGAUGUCUCCUUCA	21	0.87
ghr-miR8168-5p	AUUCAAACACAACACAGUGCA	21	0.97
ghr-miR7508-5p	CAAGAAAAGAAGUCGGGAGAG	21	0.94
ghr-miR8169-5p	CGGACUCUCAAACAGUGGAGGUA	23	0.90
ghr-miR8170-3p	CAAAUGAGUUAGGCGAGAGGU	21	1.12
ghr-miR8171-3p	UCGGGGCUUUAGCGGCGUUUUUA	23	1.12
ghr-miR8172-5p	GACGGGUGAUGGAAGUUUUUGG	22	0.96
ghr-miR8173-5p	GGAAUGGAGGAGUUGGAAAGA	21	1.40
ghr-miR8174-3p	ACAGCUUUAGAAAUCAUCCCU	21	1.00
ghr-miR8175-3p	AUGAGCUAGAAGUUGGAACUC	21	1.21
ghr-miR8176-5p	UAAGUGAAGAAAGAGGUAGGUU	22	1.16
ghr-miR8177-5p	UCAUGGUCUUUAGCGGUGUUU	21	1.34
ghr-miR8178-3p	AUGAGCUAGAAGUUGGAACUC	21	1.24
ghr-miR8179-5p	UCGGACUGGAUUUGUUGACAA	21	0.91
ghr-miR8180-5p	UGAACUUGGUAACUAUUCCCAC	22	1.16
ghr-miR8181-5p	UGAGUGGAGUUAGGAGACAAA	21	0.92
ghr-miR8182-3p	UCGCUUCCCUAAUUUGGACGA	21	0.86
ghr-miR8183-3p	GCAUCAGAGGACUCAGGCAGGU	22	1.07
ghr-miR8184-5p	UGGCACGGCUCAAUCAAAUUA	21	0.98
ghr-miR8185-5p	UUAGAAGUUAGAUUGCAUUUUG	22	1.43
ghr-miR8186-3p	UCUAACGUGUAGGGACUAAUU	21	1.22
ghr-miR8187-5p	UUAUGAUCUUUAGCGGCGUUU	21	1.38
ghr-miR8188-5p	UUGCAUGACACUACUUUAAAU	21	1.06
ghr-miR8189-3p	GUGUUUCGCGCGUGGACGACG	21	1.26

### Identification of miRNA targets in *G. hirsutum*

Identification of putative miRNA targets is an effective approach for identifying the functions of miRNAs. Plant miRNAs typically have higher sequence complementarity with their target mRNAs than do animal miRNAs (Wang et al. 2004). We search for potential silenced target genes by the novel miRNAs isolated from cotton base on transcriptome sequencing data generated for KV-1 cotton infected with the D07038 and V991 *V. dahliae* strains as described in the Materials and Methods section. We were predicting 49 putative targets (Additional file 4) for 24 novel microRNA candidates. No potential targets were identified for 13 of the novel miRNAs.

To have a deeper understand functions of microRNA, we subjected the identified target genes to gene ontology (GO) analysis (http://www.geneontology.org), a method for identifying relevant miRNA–gene regulatory networks based on biological processes and molecular functions (Ashburner & Bergman 2005). KEGG is the major public pathway-related database (Elliott et al. 2008) and it was also used to estimate the potential functions of predicted target gene candidates of the novel miRNAs. Analysis of regulatory networks and biochemical pathways help understand the biological functions of the miRNA targets. Some miRNA families had multiple target genes, which were usually different function with homologous sequences. For these target genes, binding sites of the putative miRNA were often located in the highly conserved regions. It is notable that two miRNAs, ghr-miR8156-3p and ghr-miR8170-3p, potentially target disease resistance genes such as mitogen-activated protein kinase kinase kinase 17, Nbs-lrr resistance protein, and plant viral-response family proteins. These targets include genes involved in plant–pathogen interactions, endocytosis, terpenoid backbone biosynthesis, isoquinoline alkaloid biosynthesis, primary bile acid biosynthesis, the MAPK signalling pathway, and biosynthesis of secondary metabolites. Our results may help advance understanding of the mechanism of resistance to Verticillium wilts in the upland cotton variety KV-1.

### Quantitative real time-PCR and data analyses

**Figure 2:**
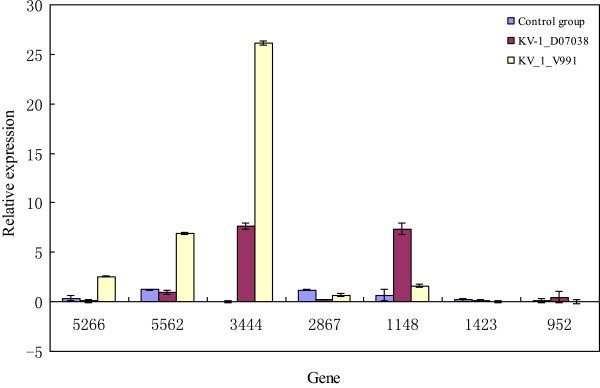
**Relative expression of seven miRNAs in different virulence Verticillium wilts of Upland cotton.**

**Figure 3:**
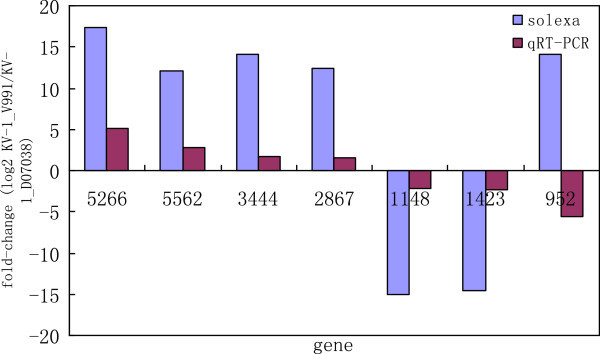
**Expression confirmation of miRNAs in** ***Gossypium hirsutum*** **derived from high throughput sequencing.** Differentially expressed miRNAs expression detected by poly(A) qRT-PCR.

Sequence counts were normalized between the datasets to account of different magnitudes raw counts. To accurate validate the deep sequencing miRNA data, we examined the correlation between normalized sequencing counts and gene expression as determined by poly (A) qRT-PCR. The expression patterns of some genes in the KV-1 plants infected by D07038 or V991 were conforming to their sequencing results. Some of the miRNA families, such as miR5266, miR5562, miR3444, and miR1148, were highly expressed, whereas miR2867, miR1423, and miR952 had relatively low level expression. A number of miRNAs were preferentially expressed in KV-1 plants infected by D07038 (e.g., miR1148) and others were preferentially expressed in KV-1 plants infected by V991 (such as miR3444) (Figure 2). There was no significant difference in miRNA levels between the Solexa and qRT-PCR data sets except for miR952 (Figure 3). We concluded that this difference was primarily due to the limitations associated with using qRT-PCR technology. The miRNA expression profiling by deep sequencing and qRT-PCR provided valuable information, however, the additional effort required to validate the data makes this approach less attractive for quantifying the effects of miRNA expression on target genes.

## Discussions

The development of high-throughput sequencing methods have greatly accelerated the identification of miRNAs in species without or with fully sequenced genomes (Peláez et al. 2012). The newly identified cotton miRNAs belonging to known miRNA families exhibited a wide range of characteristics. Previous studies have focused primarily on miRNA expression in ovules, during fiber development, and with respect to stress responses (Khan Barozai et al. 2008; Pang et al. 2009; Yin et al. 2012b). For example, miR172, miR2948, miR2949, miR2950, miR3476, miR399, miR479, and miR827 were identified during the development of ovule and fiber in upland cotton (Pang et al. 2009). In addition to miRNAs related to cotton fiber development, some miRNAs have been showed to play most vital role in response to growth stages and growth conditions. For example, ghr-miR482 regulates NBS-LRR defence genes by suppressing the miRNA-mediated gene-silencing pathway in cotton during fungal pathogen infection in cotton (Zhu et al. 2013). Liu Yang also reported that bra-miR1885 the targets protein-coding disease-resistance genes of the TIR-NBS-LRR class (Yang et al. 2013). 14 novel cotton miRNAs (miR1321 to miR1334) were identified from the *G. barbadense L*. variety ‘Hai-7124’, a Verticillium-tolerant cultivar, and the *G. hirsutum* L. variety ‘Yimian-11’, a Verticillium-sensitive cultivar, after roots were mock-infected and infected with Verticillium (Yin et al. 2012a). There have been no reports on the potential involvement of miRNAs in mechanisms of resistance to different Verticillium wilts in upland cotton varieties. Plenty of plant miRNAs have been submitted to miRBase and their biological functions have been investigated using the Illumina Genome Analyzer. Our deep sequencing data includes conserved and novel miRNAs from upland cotton based on the *G. raimondii* genome sequence. We identified 443 conserved and 37 non-conserved miRNAs from our cotton sRNA data, and also analysed the miRNAs which differentially expressed against the two *V. dahliae* strains used. Moreover, we predicted 49 potential targets of novel miRNAs to take part in plant–pathogen interactions. Our results show that miR8163, miR8165, miR8170, miR8175, and miR8178 are involved in defence responses through relevant regulatory networks, but the underlying mechanisms remain to be elucidated. Consistent with the control group, miR8163, miR8165, miR8170 and miR8175 showed significantly low level expression in our study. Similarly, because the expression level of NBS-LRR defence genes and plant viral-response family proteins were up-regulated, we can conclude that the new miRNA may involve in the regulation of the cotton in response to Verticillium wilt. The identification of these novel microRNAs and their potential target genes improves our understanding of the mechanisms regulating defense responses. We used qRT-PCR analysis to confirm the predicted known miRNAs and found that most of the expressed miRNA shave Verticillium wilt resistance characteristics. Their specific expression could provide important information about how they function. Our future work will focus on their roles in upland KV-1 cotton resistance to Verticillium wilts. This study also provides a glimpse of the abundance and diversity of sRNAs in upland cotton.

## Electronic supplementary material

Additional file 1: **Differential miRNA expression between the defoliating** ***V. dahliae*** **strains D07038 (moderate virulence) and V991 (high virulence) at the existence in the Verticillium wilt-resistant upland cotton variety KV-1.** (XLS 66 KB)

Additional file 2: **Novel miRNAs detected in the Verticillium wilt-resistant upland cotton variety KV-1.** (XLS 37 KB)

Additional file 3: **Predicted fold-back structures using precursor sequences of newly identified miRNAs in the Verticillium wilt-resistant upland cotton variety KV-1.** (DOC 96 KB)

Additional file 4: **Predicted targets for the newly identified putative miRNAs in the Verticillium wilt-resistant upland cotton variety KV-1.** (DOC 64 KB)
