# Supplementary material for: Identification of novel microRNAs in the Verticillium wilt-resistant upland cotton variety KV-1 by high-throughput sequencing
Source: Springerplus. 2014 Sep 27;3:564. doi: 10.1186/2193-1801-3-564 (PMC4190182; doi:10.1186/2193-1801-3-564)
Supplement: Supplementary file 3 — Additional file 3: Predicted fold-back structures using precursor sequences of newly identified miRNAs in the Verticillium wilt-resistant upland cotton variety KV-1. (DOC 96 KB) [file 40064_2014_1265_MOESM3_ESM.doc]

(1) ghr-miR8156-5p: AAACUAUUCCUGGCUGAUUCG

ghr-miR8156-3p: AAUUAGCCAGGAAUCGUUUGA

10 20 30 40 50

AU| C U CACCAGU UU UUAGUU

GAAUAA CAAAC AUUCCUGGCUGAUUCG UA CA U

CUUAUU GUUUG UAAGGACCGAUUAAGC AU GU A

CU^ A C CACU--- UU UACUUG

90 80 70 60

(2) ghr-miR7513-3p: AAUCAGCCAGGAAUCGUUUGA

ghr-miR7513-5p: AAACUAUUCCUGGCUGAUUCG

10 20 30 40 50

GCAU| C U CACCAGU UU UUAGUU

GAAUAA CAAAC AUUCCUGGCUGAUUCG UA CA U

CUUAUU GUUUG UAAGGACCGACUAAGC AU GU A

UACU^ A C CACU--- UU UACUUG

100 90 80 70 60

(3) ghr-miR8157-5p: AAGGCAAAGGAAGAAAAAGAGUG

ghr-miR8157-3p: CUCUUUUUUUCCUGCCUUGC

10 20 30 40 50

UC| A AA A GAU UG -- AAA

AGAUCGG AAGGCA GGAAGAAAA GAGUGAAA U G GUUUUGAG A

UCUAGUC UUCCGU CCUUUUUUU CUCACUUU G C CAAAACUU U

UU^ G -- - --- GU AG AAC

100 90 80 70 60

(4) ghr-miR8158-3p: AAGGGAGAACCUAGAUUCAUU

ghr-miR8158-5p: AAUGAAUCUAGGUUCUCUCUU

10 20 30 40 50 60

UCU- AC - C --| CAUUUUUU CU

AUGA CUUUU CUGGG AAUGAAUCUAGGUUCUCUCU UGCUC CAUAUUUUC \

UACU GAAAA GACCC UUACUUAGAUCCAAGAGGGA GCGAG GUAUAAAAG U

CAAC -- C A AU^ AACUUCU- UG

. 120 110 100 90 80 70

(5) ghr-miR8159-5p: AAUGGAGGAGUUGGAAAGAUU

ghr-miR8159-3p: UCUUUCCAAUUCCUCCCAUUCC

10 20 30 40 50

CC---| UU UU - GA UUCUCUG

GU CUGGAAAUU CGGAAUGG AGGAGUUGGAAAGAUUUU AUU U

CA GACCUUUAG GCCUUACC UCCUUAACCUUUCUAAAA UAA A

UCCUU^ C- UC C AG UAAUUUU

110 100 90 80 70 60

(6) ghr-miR8160-5p: ACAGCUUUAGAAAUCAUCCCU

ghr-miR8160-3p: GGAUGAUUUCUAAAGCUCUAG

10 20 30 40 50

ACU C UA-| AAAUAUAUA

GUUAUCUA AGCUUUAGAAAUCAUCCCUUUUU GAUGUGU A

CAAUAGAU UCGAAAUCUUUAGUAGGGAAAGA UUACACG A

UUG C UCG^ AAGUUAAAU

100 90 80 70 60

(7) ghr-miR8161-3p: GUGGAUUAAAAUUUUGGUUGG

ghr-miR8161-5p: ACCCAAAGUUUUAAUCCACUA

10 20 30 40 50 60 70

C C .-UA CG GACA- G GA-----| GGA

UCCUUUC--CCA CCAAAGUUUUAAUCCACUACAG CCU GACAU UUUA UGC UAUACACCUA \

GGGAAAG GGU GGUUUUAAAAUUAGGUGAUGUU GGA UUGUA AGAU ACG AUAUGUGGAU U

U \ U \ -- AU AUAAA A AUAUGUG^ AUU

. 150 140 110 100 90 80

120

-- G

AUGCC U

UAUGG U

UG A

130

160

CU----- U

UUGGA C

AACCU A

AUCUGUC A

180 170

(8) ghr-miR8162-5p: ACUUGCCUGCAUCUUUCAAAGA

ghr-miR8162-3p: UUUGUGAGGGUGCCAGGCAGUCA

10 20 30 40 50

-| UGA U - - G AAAAUC GUA A

GCCAA UGACU GCCUG CAUCUUU CAAAGAG UUC UCU UCA C

CGGUU ACUGA CGGAC GUGGGAG GUUUCUC AAG AGA GGU C

U^ CC- - C U G ACCA-- AAA A

100 90 80 70 60

(9) ghr-miR8163-3p: GGUUGCUUACUUCUCUUCUGU

ghr-miR8163-5p: AGAAGAGAGUGAGCACUCAC

10 20 30 40

AG| A - CU A GC UU AA

AG UUGACAGAAGAGA GUGAGCA CAC G AA GUAUGC \

UC GGCUGUCUUCUCU CAUUCGU GUG C UU CAUACG A

UA^ - U UG C UA UC GA

90 80 70 60 50

(10) ghr-miR8164-3p: CCUAAUAAGGAUGAUGUCUCA

ghr-miR8164-5p: AGACAUCAUCCUUAUCAAGAA

10 20 30 40 50

U G CAA .-UGCA| UUUCAAUU

CAAUCAAA AUGAGACAUCAUCCUUAU GAAACUG GGAUUCAA A

GUUAGUUU UACUCUGUAGUAGGAAUA CUUUGAC UUUAAGUU A

- A AUC \ ----^ UUGAAACA

230 220 210 200 70 60

80 90 100 110 120

CUUCUAUA AA--- UCU- .-A A UU U

AAAGGAGGUG AGAAG AAAUG AGC UUGAAG GGAU U

UUUCCUUUAU UUUUC UUUAU UUG AACUUC CUUG A

CAUCA--- AAAUG UCUC \ - - CU A

190 180 170 140 130

150

AUUU AA

CUUA A

GAAU U

U--- CU

160

(11) ghr-miR8165-3p: UCCAUAUUUCACUAUCUCUUA

ghr-miR8165-5p: AGAGAUAGUGCAAUAUGGAGG

10 20 30 40 50 60

AAUCUGAU CU----- C -| A AU

UGUCUUU AUAAGAGAUAGUG AAUAUGGAGGCUUG AGCA AGUGAAA G

ACAGAAG UAUUCUCUAUCAC UUAUACCUCUGAAC UUGU UCAUUUU A

CUUGC--- UAUUCCC U U^ A AU

120 110 100 90 80 70

(12) ghr-miR8166-3p: CACAGGGACAAUACCUUCUAC

ghr-miR8166-5p: AGAGGGUAUUGUUUUCGUGGG

10 20 30 40 50 60 70 80 90 100

U ACAAC UC UU U C A C- A C A - .-AUUG| UC

CACUCCCUCA AGAGGGUAUUGUUU GUGGGGGGU UGUUUCCCAU UU UGA GAGAGUC AAAGGG UUCA GGA UUU AGGA CC G

GUGAGGGAGU UCUUCCAUAACAGG CACCCCUCA ACAGAGGGUA AA ACU CUCUCGG UUUCCC AAGU CCU AAA UCCU GG A

- CA--- GA -- U A C UU - A C U \ ----^ UC

240 230 220 210 200 190 180 170

110 120 130

AAGA U A AGCU G AAA

GA ACG UUUGCA CAC GG \

CU UGU AAACGU GUG CU G

A--- - A AUU- A GUG

160 150 140

(13) ghr-miR8167-5p: AGCUUUAGAAAUCAUCCCUU

ghr-miR8167-3p: GGGAUGAUUUCUAAAGCUGU

10 20 30 40 50

C G C-| CA CUUCAAUUUA

GUUAUCUA AGCUUUAGAAAUCAUCCCUUU UAG AUGUG U

CAAUAGAU UCGAAAUCUUUAGUAGGGAAA AUC UACAC U

A G AA^ -- AUUUAUAUAU

100 90 80 70 60

(14) ghr-miR7495a-3p: UUACUUUAGAUGUCUCCUUCA

ghr-miR7495a-5p: AGGGAAACAUCUAAAGUAAAC

10 20 30 40 50 60 70 80 90

UUCA| U A CGA AA U AU UAA AACUUAGUGUUU CA- AUU

ACAGC UGAGGGA ACAUCUAAAGUAAA CG CGAACU CC UGA CCGAUG GAAGGAAG AGAUC \

UGUCG ACUUCCU UGUAGAUUUCAUUU GU GCUUGA GG ACU GGCUGC CUUCUUUU UUUGG U

UGUC^ U C UUG CC U -- --- ------------ UAC CCC

160 150 140 130 120 110 100

(15) ghr-miR8168-5p: AUUCAAACACAACACAGUGCA

ghr-miR8168-3p: CACUGUGUUGUGUUUGAAUCC

10 20 30 40 50 60 70 80 90

A C A .-C C G C .-CUGGA C G A--------| G

UUC UUUGGAUUCAAACACAACACAGUGC UUGAAA GUUUCAC GUACUG AG UGA GCUUUUAGCA CAGU GAGAU ACGCU U

AAG AAACCUAAGUUUGUGUUGUGUCACG AACUUU UAGAGUG CGUGGU UC ACU CGAAGGUCGU GUUA CUCUG UGUGA C

G A A \ - A G - \ ----- - - CAAAAACGA^ C

220 210 200 170 160 120 110 100

130 140

AAGACAUUCAAACA C

GC \

CG A

UAA----------- A

150

---------- AA

UG \

AC A

UAAAAAUAGA UA

190 180

(16) ghr-miR7508-5p: CAAGAAAAGAAGUCGGGAGAG

ghr-miR7508-3p: UUCCCGGCUUCUUUUCUUGCU

10 20 30 40 50

UGGCA--| A C C CCCUUUA

GGAAGCAAGAAAAGAAGUCGGGAGAG AUUUU GAU GGUU G

CUUUCGUUCUUUUCUUCGGCCCUUUC UAAAG CUA UCAA C

UAAUUUG^ C C U AACUCUU

. 100 90 80 70 60

(17) ghr-miR8169-5p: CGGACUCUCAAACAGUGGAGGUA

ghr-miR8169-3p: CCUCCAUUGUUUGAGGGUCUGAU

10 20 30 40 50 60 70 80 90 100

AGCA A--- AA- - A .-GUUUAG GAA UU -----| C A GAUU A

GUC AUCGGACUCUCAAACAGUGGAGGUAUUGUGCU GGUG UCU UGCUCA CC GGU UU GUCUUU AACUCCG GGA CC A

CAG UAGUCUGGGAGUUUGUUACCUCCAUAAUACGG CUAC AGA AUGGGU GG CCA AA CAGGGA UUGAGGC CUU GG C

UUCA AUGC ACC A - \ ------ AG- CU UCUGU^ U A ---- G

250 240 230 220 210 200 130 120 110

140 150 160

A- UAGCAAUU CUUA U

AAUGUU GGC GUGGGUU \

UUACAG CCG UACCCAA U

GA UAAAUUC- ---- G

190 180 170

(18) ghr-miR8170-3p: CAAAUGAGUUAGGCGAGAGGU

ghr-miR8170-5p: CUCUGGUUUGACUCAUUUGUA

10 20 30 40 50 60

- G UAUU CG .-ACAA| CAUU

UUCAAUGAACCUCU GUUUGACUCAUUUGUAAUAAU UUCGCU UUGGAU CCG \

AAGUUACUUGGAGA CGGAUUGAGUAAACAUUGUUA AAGCGA AACUUA GGU U

G G UAAC -- \ ----^ AAAU

. 160 150 140 130 120 70

80

AAAA------------------- AAUA

CUGU A

GACA U

AUUAGAACAAGAAACGAAAAGAA CUCC

110 100 90

(19) ghr-miR8171-3p: UCGGGGCUUUAGCGGCGUUUUUA

ghr-miR8171-5p: GAAGCGCCGCUAAUGCUCAGGGU

10 20 30 40 50 60 70

U| UUA U A A AAA CCA U C

GGUGUUUU AGAAGCGCCGCUAA GCUC GGGUUUUAG GGUGCUUUUU AAGCG CUAA GAU \

CCGCAAAG UUUUUGCGGCGAUU CGGG CUCAAAAUC CCGCGAAAAA UUUGC GAUU CUG G

-^ UAA U G A GUC AGC U G

140 130 120 110 100 90 80

(20) ghr-miR8172-5p: GACGGGUGAUGGAAGUUUUUGG

ghr-miR8172-3p: AAACAUUUCCGCUGCUUCUCUC

10 20 30 40 50 60 70 80

-| GU CG GA U A - CGC C- CUCUUUCCCAUCU U A

GAGGG AGAGA GGU UGGAAGU UUUGGAG UGGGUGGA UUGGCAAAA UC CUUC AUUGUAU UGU A

UUCCC UCUCU UCG GCCUUUA AAACCUU ACCCGCCU GACCGUUUU AG GAAG UAACGUA AUA U

G^ UG CU UC C - C --- UU ------------- U A

160 150 140 130 120 110 100 90

(21) ghr-miR8173-5p: GGAAUGGAGGAGUUGGAAAGA

ghr-miR8173-3p: UUUCCAAUUCCUCCCAUUCCAC

10 20 30 40 50

U UUC -| GA UUCUCUG

CUGGAAAUU GGAAU GGAGGAGUUGGAAAGAUUUU AUU U

GACCUUUAG CCUUA CCUCCUUAACCUUUCUAAAA UAA A

- UCA C^ AG UAAUUUU

100 90 80 70 60

(22) ghr-miR8174-3p: ACAGCUUUAGAAAUCAUCCCU

ghr-miR8174-5p: GGAUGAUUUCUAAAGCUUUGG

10 20 30 40

AUAAC U U | A AU

AAGGAA GGAUGAUUUCUAAAGCU UGGAUA--GCUUU UC \

UUUUUU CCUACUAAAGAUUUCGA AUCUAU CGAAG AG G

GUAGA C C \ ^ G UA

. 140 130 120 50

60 70 80

AA-- ACC - GU GU

UGC GAGUU CAAUAUU CAU \

AUG UUUAA GUUAUAA GUG A

UGUC AUA C AG GU

110 100 90

(23) ghr-miR8175-3p: AUGAGCUAGAAGUUGGAACUC

ghr-miR8175-5p: GUUCCAACUUCUAACUCAUUU

.-G| GUU

AGGUU \

UCCAA U

\ -^ AUA

20 30 40 50 60 70 80

AAUUUGUUU- A UGU AUAUA--- U UCU

GAGUUCCAACUUCUA CUCAUUUUCAGCCU UUCUUUCGA GU GUUU \

CUCAAGGUUGAAGAU GAGUAAAAGUUGGA AAGAAAGUU CA CAAA A

AACCAAAAAU C UU- AACGAACG C UCC

150 140 130 120 110 100 90

(24) ghr-miR8176-5p: UAAGUGAAGAAAGAGGUAGGUU

ghr-miR8176-3p: CCAGCCUUUUCUUCACUUAGU

10 20 30 40 50 60 70

- A A .-AGAA| U GAGUUUA AG U A A

GAAGGACAACUAAGUGAAG AAGAGGU GGUUUGAAGA GAUGG AAGCU AG GAA GGUGA CUU A

CUUCCUGUUGAUUCACUUC UUUUCCG CCAAACUUCU CUGCU UUUGA UC CUU CUACU GAG U

C - A \ ----^ - ------- CU - A U

160 150 140 100 90 80

110

AC--- CC

AUGAA U

UACUU U

AGAUU CU

130 120

(25) ghr-miR8177-5p: UCAUGGUCUUUAGCGGUGUUU

ghr-miR8177-3p: GCGCCGCUAAAGGUCAUGGUC

10 20 30

-| U GU GUG

CC CUAAAGAUCAUG CUUUAGCGGUGUUU \

GG GAUUUCUGGUAC GAAAUCGCCGCGAA A

C^ C UG AAG

. 60 50 40

(26) ghr-miR8178-3p: AUGAGCUAGAAGUUGGAACUC

ghr-miR8178-5p: UCCAACUUCUAACUCAUUUUC

10 20 30 40 50 60 70

UA--- AAUUUG - A UGU AUAUA---| U UCU

AACCU UUU GAGUUCCAACUUCUA CUCAUUUUCAGCCU UUCUUUCGA GU GUUU \

UUGGA AAA CUCAAGGUUGAAGAU GAGUAAAAGUUGGA AAGAAAGUU CA CAAA A

AAGUA AACCAA U C UU- AACGAACG^ C UCC

150 140 130 120 110 100 90 80

(27) ghr-miR8179-5p: UCGGACUGGAUUUGUUGACAA

ghr-miR8179-3p: GUCAACAAAUACCAGUUUGAGC

10 20 30 40 50 60

-------| AUU GU UG A - --- U GAAGC AA

CAGU CCG GUCU G UCGGACUGG AUUUGUUGAC AAA GCAUUUUU AGAU \

GUCG GGU UAGA C AGUUUGACC UAAACAACUG UUU CGUAAAAA UCUA C

ACUUAGA^ --- -- GU G A ACC U ----- AA

120 110 100 90 80 70

(28) ghr-miR8180-5p: UGAACUUGGUAACUAUUCCCAC

ghr-miR8180-3p: GGGAACAAUUGCCAAGUUCAGG

10 20 30 40 50

AGUA CUA A .-AAAG| C

AGUCUCUGAACUUGGUAA UUCCCACGUU GGGCUUUAACUU GUUUU A

UUAGGGACUUGAACCGUU AAGGGUGUAA CCCGGAGUUGAA UAAAG A

UUAA AAC C \ ----^ G

. 130 120 110 100 60

70

A-------- CA

UCAGG C

GGUUC U

AAGAAACCA AU

90 80

(29) ghr-miR8181-5p: UGAGUGGAGUUAGGAGACAAA

ghr-miR8181-3p: UGUCUCCCAACUCCGCUCACA

10 20 30 40 50 60 70 80 90

U GG A A C UUAA- AA - - C GG C UG -----| U A

CUU UCU UGAGUGGAGUU GGAGACAAAA CUUC GGA AAUG UGGAC UG UGAGGGGUU UCAGCUC CGG GGCG UCU AAG A

GAA AGA ACUCGCCUCAA CCUCUGUUUU GAGG CCU UUGC GUCUG AC AUUCCCCGG GGUCGAG GUU UUGU AGG UUC G

U A- C C - UACCC G- G U A GU C GU UUUUA^ - U

190 180 170 160 150 140 130 120 110 100

(30) ghr-miR8182-3p: UCGCUUCCCUAAUUUGGACGA

ghr-miR8182-5p: UGCCAAAUCAGGGAAGCGAAA

10 20 30 40 50 60 70

AAU--| AUUU - UG C AGGU CA C C UU GUAGUU

GGAAC GG AAAAUU CCAAAU AGGGAAGCGAA GUUUG GG AG AGAGU UGAGG \

CCUUG UC UUUUAG GGUUUA UCCCUUCGCUU UAGAC CC UC UCUCA ACUCU U

UACUU^ CGU- A CA A AU-- -- - A CU ACUACU

140 130 120 110 100 90 80

(31) ghr-miR8183-3p: GCAUCAGAGGACUCAGGCAGGU

ghr-miR8183-5p: UGCCUGGCUCCCUGAAUGCCA

10 20 30 40 50 60

----| AACAAU AAU GC - A U U- U AUAA U

AUGA GGA GAUCUGCCUG UCC CUGA UGCCAACUA GGA GG GUCAA GCGG G

UACU CCU CUGGACGGAC AGG GACU ACGGUUGAU CCU CC CGGUU UGUC U

UUUC^ AUUUUU AU- UC A - - UU - GU-- U

130 120 110 100 90 80 70

(32) ghr-miR8184-5p: UGGCACGGCUCAAUCAAAUUA

ghr-miR8184-3p: AUUUGAUUGAGCCAUUCCAAC

10 20 30 40

-| U A CAC AGA CACUAA

CUGG GAU UUGG GGCUCAAUCAAAUUAAA CC \

GACC CUA AACC CCGAGUUAGUUUAGUUU GG A

U^ C C UUA AAA AACUCA

. 80 70 60 50

(33) ghr-miR8185-5p: UUAGAAGUUAGAUUGCAUUUUG

ghr-miR8185-3p: AAAUACAAUUUAACUCUUAAUA

10 20 30 40 50

- C-- GA C A - .-CAAAA| GA

GAC CUUGUAUUA AGUUAGAUUG AUUUUGCUC UUC ACU AAGGGGC A

CUG GAACAUAAU UCAAUUUAAC UAAAACGAG AAG UGA UUCCCCG U

C CGA UC A A A \ -----^ AU

340 330 320 310 300 60

70 80 90 100 110 120 130 140 150

GCAUAAGAUAAAA C A .-AUUUUU C A -------------- .-CAAACAGA CUA

GAG AAA UGAUC GUUAAAAAUUUCAUCAAUUU UAC GUUAAAAACU AGUAUGA AUAA G

CUC UUU AUUAG UAAUUUUUAAAGUAGUUAAA AUG UAAUUUUUGA UCGUACU UAUU A

AAUUUUUG----- A A \ ------ A A CCAGAUAUAUAUAA \ -------- AGC

290 280 240 230 220 210 200 190 160

170

AC- GU

AUGGU G

UAUCA C

CUA AU

180

250

-------------- CU

UAAAGGGC A

AUUUUUCG U

GUAUAUACAUUAUA UU

270 260

(34) ghr-miR8186-3p: UUAGUCUUUGUCCGUUAGAUC

ghr-miR8186-5p: UCUAACGUGUAGGGACUAAUU

10 20 30 40 50 60 70 80 90 100

A UC C A G UU G - UU .-UUUAGCG| C

AAAAUGGGCAAAUUAGUCUUUG CGUUAGAU AAAGAGC AA UGGUUCUU UGU UAAAAA UUCAUUCG UUUAUUGUUAAAA UGGUUGA G

UUUUACCCGUUUAAUCAGGGAU GCAAUCUA UUUCUCG UU AUUAGGAA ACA AUUUUU AAGUAGGU AGAUGAUAAUUUU ACCAAUU A

- GU U A G U- - A U- \ -------^ A

240 230 220 210 200 190 180 170 110

120

.-AACA C UG

GUUACA G \

CGAUGU C C

\ ---- A AC

130

140

AUA-- GAC

UUCU U

AAGA U

UGACC ACA

160 150

(35) ghr-miR8187-5p: UUAUGAUCUUUAGCGGCGUUU

ghr-miR8187-3p: GCGCUGCUAAAGGUCAUGAUC

10 20 30

UC| AG GUG

GCUAAA UUAUGAUCUUUAGCGGCGUUU \

CGAUUU AGUACUGGAAAUCGUCGCGAA A

GA^ CU GAU

60 50 40

(36) ghr-miR8188-5p: UUGCAUGACACUACUUUAAAU

ghr-miR8188-3p: UUAAAGUAGUGUCCUGCAAAC

10 20 30 40

GCA| CC U AUUAA AUUA

UGUG UUUGCA GACACUACUUUAA CAUU \

ACAC AAACGU CUGUGAUGAAAUU GUAA A

CAA^ UC C AACA- CAAA

80 70 60 50

(37) ghr-miR8189-3p: GUGUUUCGCGCGUGGACGACG

ghr-miR8189-5p: UUGUCCACGCGCGACACGCAC

10 20 30 40 50

A-- -| A U C - UUUGU U

AAUGAC UGUUGA UUU UGUUGUCCACGCGCGA ACGCAC UGCA GCUUU \

UUACUG ACAAUU AAA GCAGCAGGUGCGCGCU UGUGUG AUGU CGAAA U

AUA A^ - U U U UU--- C

110 100 90 80 70 60
