# Supplementary material for: Identification of novel microRNAs in the Verticillium wilt-resistant upland cotton variety KV-1 by high-throughput sequencing
Source: Springerplus. 2014 Sep 27;3:564. doi: 10.1186/2193-1801-3-564 (PMC4190182; doi:10.1186/2193-1801-3-564)
Supplement: Supplementary file 4 — Additional file 4: Predicted targets for the newly identified putative miRNAs in the Verticillium wilt-resistant upland cotton variety KV-1. (DOC 64 KB) [file 40064_2014_1265_MOESM4_ESM.doc]

| ID | miRNA_id | Target gene | gene | Biological Process |
| --- | --- | --- | --- | --- |
| KV-1_D07038-m0113 | ghr-miR8156-5p | No hit was found | - | - |
| KV-1_D07038-m0180 | ghr-miR7513-3p | Methionyl-trna synthetase | Unigene32430_KV-1 | Aminoacyl-tRNA biosynthesis |
| KV-1_D07038-m0273 | ghr-miR8157-5p | Phosphatidic acid phosphatase (PAP2) family protein | Unigene12662_KV-1  Unigene12663_KV-1 | - |
| KV-1_D07038-m0100 | ghr-miR8158-3p | Cullin family | Unigene12909_KV-1 | Ubiquitin mediated proteolysis |
| KV-1_D07038-m0413 | ghr-miR8159-5p | 3-deoxy-d-arabino-heptulosonate-7-phosphate synthase  Ammonium transporter | Unigene26909_KV-1  Unigene11750_KV-1  CL3798.Contig1_KV-1 | Phenylalanine, tyrosine and tryptophan biosynthesis  Metabolic pathways  Protein processing in endoplasmic reticulum |
| KV-1_D07038-m0218 | ghr-miR8160-5p | Allantoinase  Pho1-like protein  pentatricopeptide (PPR) repeat-containing protein  Binding protein  tRNA-binding arm; t-snare | Unigene5719_KV-1  Unigene32108_KV-1  Unigene32109_KV-1  Unigene45208_KV-1  Unigene45209_KV-1  Unigene45210_KV-1  Unigene47232_KV-1 | Purine metabolism  Metabolic pathways  Microbial metabolism in diverse environments |
| KV-1_D07038-m0007 | ghr-miR8161-3p | Chromatin remodeling complex subunit  CBS domain-containing protein | Unigene11202_KV-1  Unigene11203_KV-1  Unigene18746_KV-1 | - |
| KV-1_V991-m0231 | ghr-miR8162-5p | Myosin XI  30s ribosomal protein S1 | Unigene7812_KV-1  Unigene10824_KV-1 | Homologous recombination  Ribosome |
| KV-1_D07038-m0494 | ghr-miR8163-3p | Remorin  AP-4 complex subunit epsilon  Transcription initiation factor  predicted protein  Cytochrome p450 | Unigene11386_KV-1  Unigene15874_KV-1  Unigene34782_KV-1  Unigene39032_KV-1  Unigene41826_KV-1  Unigene57378_KV-1 | Lysosome  RNA transport  mRNA surveillance pathway  Metabolic pathways  Microbial metabolism in diverse environments  Aminobenzoate degradation  Biosynthesis of secondary metabolites  Limonene and pinene degradation  Bisphenol degradation  Polycyclic aromatic hydrocarbon degradation |
| KV-1_V991-m0132 | ghr-miR8164-3p | No hit was found | - | - |
| KV-1_D07038-m0023 | ghr-miR8165-3p | Nbs-lrr resistance protein  Cytokinin oxidase  Cc-nbs-lrr resistance protein | Unigene47520_KV-1  Unigene49399_KV-1  Unigene67353_KV-1 | Plant-pathogen interaction  Zeatin biosynthesis |
| KV-1_D07038-m0284 | ghr-miR8166-3p | No hit was found | - | - |
| KV-1_D07038-m0219 | ghr-miR8167-5p | Allantoinase  OBP3-responsive protein 1  Pentatricopeptide (PPR) repeat-containing protein  Binding protein  Plastid hexose transporter | Unigene5719_KV-1  Unigene22132_KV-1  Unigene38936_KV-1  Unigene43769_KV-1  Unigene45209_KV-1  Unigene45210_KV-1  Unigene47973_KV-1  Unigene52485_KV-1  Unigene69991_KV-1  CL2685.Contig1_KV-1  CL3058.Contig1_KV-1 | Purine metabolism  Metabolic pathways  Microbial metabolism in diverse environments  RNA transport |
| KV-1_D07038-m0199 | ghr-miR7495a-3p | Pentatricopeptide repeat-containing protein | Unigene61075_KV-1  Unigene70954_KV-1 | Aminoacyl-tRNA biosynthesis |
| KV-1_D07038-m0237 | ghr-miR8168-5p | Similar to transducin family protein / WD-40 repeat family protein | Unigene33003_KV-1  Unigene33004_KV-1 | Spliceosome  Basal transcription factors  Ribosome biogenesis in eukaryotes |
| KV-1_D07038-m0128 | ghr-miR7508-5p | 2-oxoglutarate-dependent dioxygenase  Multidrug resistance protein ABC transporter family  F-box/ankyrin repeat protein SKIP35  Metal-nicotianamine transporter YSL1 | Unigene22605_KV-1  Unigene33491_KV-1  Unigene49833_KV-1  Unigene62849_KV-1 | Diterpenoid biosynthesis  Biosynthesis of secondary metabolites  ABC transporters  Bile secretion |
| KV-1_D07038-m0332 | ghr-miR8169-5p | No hit was found | - | - |
| KV-1_V991-m0422 | ghr-miR8170-3p | S-locus-like receptor protein kinase | Unigene43443_KV-1 | Plant-pathogen interaction  Leishmaniasis  Plant hormone signal transduction  Chagas disease (American trypanosomiasis)  Toll-like receptor signaling pathway  Neurotrophin signaling pathway  Toxoplasmosis  Apoptosis  Measles |
| KV-1_D07038-m0060 | ghr-miR8171-3p | No hit was found | - | - |
| KV-1_D07038-m0016 | ghr-miR8172-5p | No hit was found | - | - |
| KV-1_D07038-m0499 | ghr-miR8173-5p | 3-deoxy-d-arabino-heptulosonate-7-phosphate synthase 02  mRNA adenosine methylase  Polyphenol oxidase 1  Oligopeptide transporter OPT family  Outer envelope protein  Sec24-like transport protein  Ribosomal protein L35  Polyphenol oxidase | Unigene11750_KV-1  Unigene26909_KV-1  Unigene49428_KV-1  Unigene55686_KV-1  Unigene57040_KV-1  Unigene57041_KV-1  Unigene57042_KV-1  Unigene57043_KV-1  CL3742.Contig1_KV-1  CL730.Contig1_KV-1  CL4175.Contig1_KV-1  CL1012.Contig1_KV-1 | Protein processing in endoplasmic reticulum  Phenylalanine, tyrosine and tryptophan biosynthesis  Metabolic pathways  Biosynthesis of secondary metabolites  Isoquinoline alkaloid biosynthesis  Tyrosine metabolism |
| KV-1_V991-m0088 | ghr-miR8174-3p | Allantoinase  Similar to lysine decarboxylase-like protein  Tetratricopeptide-like helical domain containing protein  Pentatricopeptide (PPR) repeat-containing protein  Binding protein  tRNA-binding arm; t-snare |  | Purine metabolism  Metabolic pathways  Microbial metabolism in diverse environments |
| KV-1_D07038-m0058 | ghr-miR8175-3p | Mitogen-activated protein kinase kinase kinase 17  Leucine-rich repeats | Unigene9429_KV-1  Unigene66172_KV-1 | Plant-pathogen interaction  MAPK signaling pathway - yeast |
| KV-1_D07038-m0313 | ghr-miR8176-5p | No hit was found | - | - |
| KV-1_V991-m0296 | ghr-miR8177-5p | Uncharacterized protein | Unigene64272_KV-1  Unigene64273_KV-1  Unigene64274_KV-1  Unigene64275_KV-1 | - |
| KV-1_V991-m0045 | ghr-miR8178-3p | Plant viral-response family protein  Mitogen-activated protein kinase kinase kinase 17  Leucine-rich repeats | Unigene9429_KV-1  Unigene66172_KV-1 | Plant-pathogen interaction  MAPK signaling pathway - yeast |
| KV-1_D07038-m0456 | ghr-miR8179-5p | No hit was found | - | - |
| KV-1_D07038-m0330 | ghr-miR8180-5p | No hit was found | - | - |
| KV-1_V991-m0030 | ghr-miR8181-5p | Myosin XI  F-box family protein  Alternative oxidase  Uncharacterized protein | Unigene14241_KV-1  Unigene18603_KV-1  Unigene18604_KV-1  Unigene35666_KV-1  Unigene50857_KV-1 | Homologous recombination  Cell cycle - yeast |
| KV-1_D07038-m0377 | ghr-miR8182-3p | No hit was found | - | - |
| KV-1_V991-m0490 | ghr-miR8183-3p | Glutamyl-tRNA reductase 1  Thiamine-phosphate pyrophosphorylase | Unigene26970_KV-1  Unigene26971_KV-1  Unigene50242_KV-1 | Porphyrin and chlorophyll metabolism  Metabolic pathways  Biosynthesis of secondary metabolites |
| KV-1_V991-m0270 | ghr-miR8184-5p | Pentatricopeptide repeat domain  Hit-type zinc finger family protein | - | - |
| KV-1_D07038-m0341 | ghr-miR8185-5p | No hit was found | - | - |
| KV-1_V991-m0472 | ghr-miR8186-3p | No hit was found | - | - |
| KV-1_V991-m0428 | ghr-miR8187-5p | Nucleolar complex protein 2-like protein  Armadillo repeat-containing protein | Unigene1196_KV-1  Unigene37648_KV-1 | Regulation of autophagy |
| KV-1_D07038-m0472 | ghr-miR8188-5p | Polyphenol oxidase | Unigene55686_KV-1 | Isoquinoline alkaloid biosynthesis  Tyrosine metabolism  Metabolic pathways  Biosynthesis of secondary metabolites |
| KV-1_V991-m0452 | ghr-miR8189-3p | No hit was found | - | - |
